# Supplementary material for: Savinin Triggers Programmed Cell Death of Ray Parenchyma Cells in Heartwood Formation of Taiwania cryptomerioides Hayata
Source: Plants (Basel). 2023 Aug 23;12(17):3031. doi: 10.3390/plants12173031 (PMC10490442; doi:10.3390/plants12173031)
Supplement: Supplementary file 1 [file plants-12-03031-s001.zip › plants-2498107-supplementary.pdf]

Supporting Information

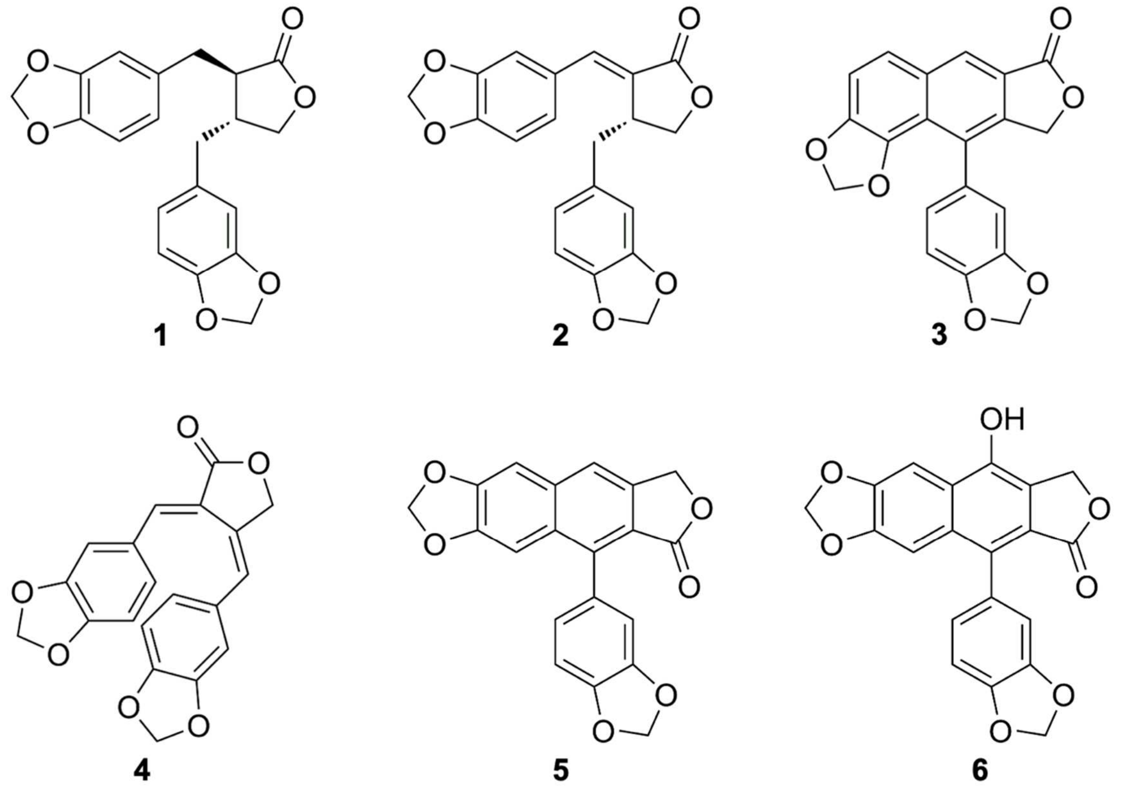

1 2 3 4 5 6

Figure S1. The structures of 6 lignans. 1, hinokinin; 2, savinin; 3, helioxanthin; 4, taiwanin A; 5, taiwanin C; 6, taiwanin E.

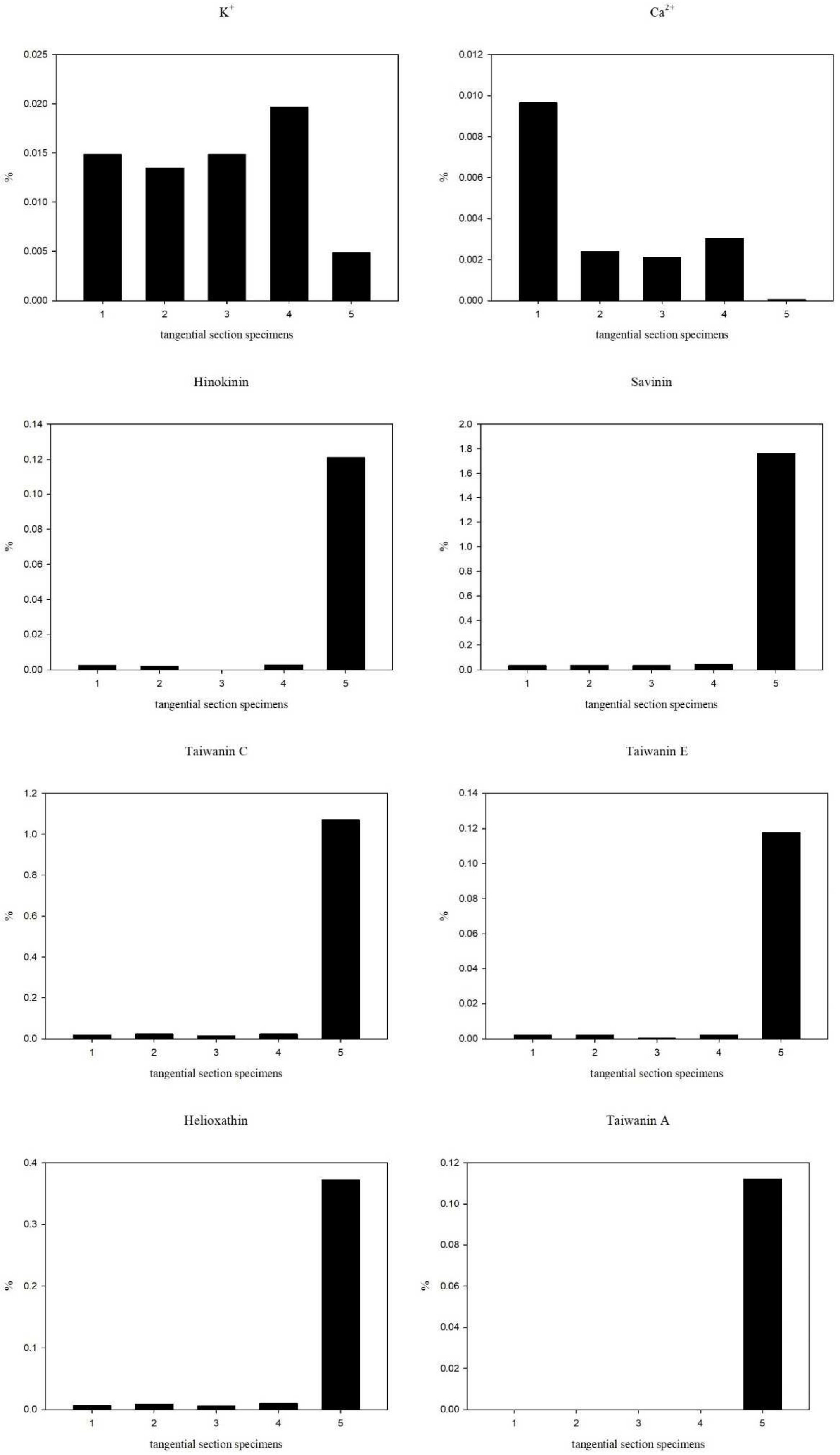

Figure S2. The quantification of K^+, Ca^{2+}, hinokinin, savinin, taiwanin C, taiwanin E,

helioxanthin and taiwanin A. That dividing the intensity of the signal by the total ion intensity.

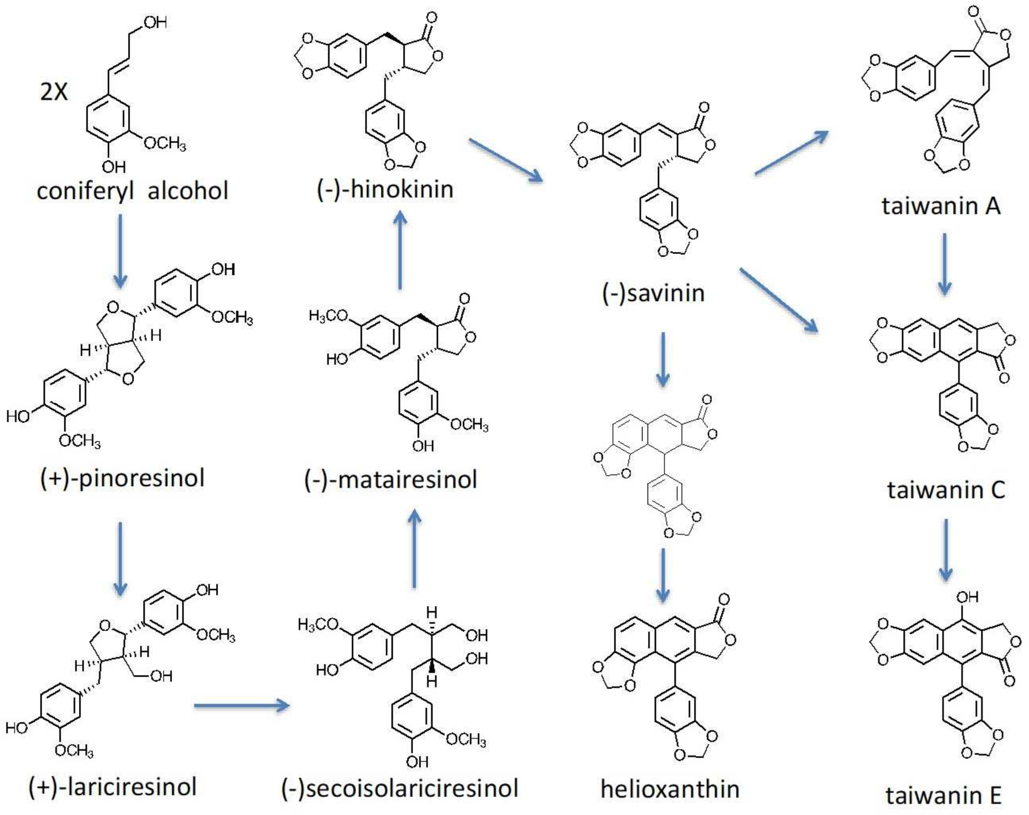

Figure S3. The biosynthetic pathway of lignans in Taiwania.

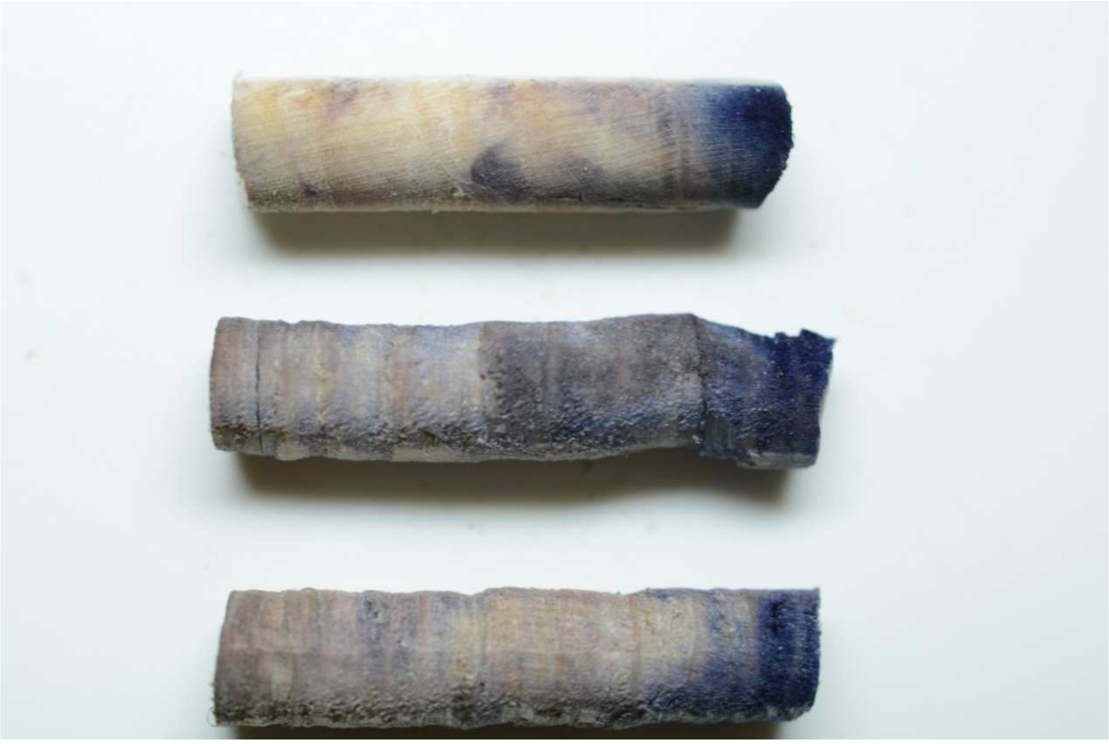

Figure S4. The wood core of Taiwania in sapwood (left) to heartwood (right) after treatment with 6 mM NBT. The formation of insoluble dark blue diformazan came from the reduction of NBT by the presence of ROS.

Table S1. Genes involved in inducing type of programmed cell death.

Type of induced PCD	Gene annotation	Gene description	FPKM (DX)	FPKM (SW)	FPKM (SW)
Developmentally-induced	AT3G45010	scpl48__serine carboxypeptidase-like 48	5.82E+01	3.74E+01	5.95E+01
	AT4G04460	Saposin-like aspartyl protease family protein	2.94E+02	2.19E+02	3.18E+02
	AT1G11190	BFN1_ENDO1__bifunctional nuclease i	1.36E-01	1.01E+01	4.38E+01
	AT1G26820	RNS3__ribonuclease 3	8.21E-03	8.36E-01	9.70E-03
	AT3G56170	CAN__Ca-2+ dependent nuclease	1.01E+00	2.41E-01	8.22E+00
	AT3G21550	AtDMP2_DMP2__DUF679 domain membrane protein 2	1.72E+00	4.80E+02	6.54E-01
	AT5G53590	SAUR-like auxin-responsive protein family	3.41E-01	8.96E+01	3.49E+01
	AT5G13820	ATBP-1_ATBP1_ATTBP1_HPPBF-1_TBP1__telomeric DNA binding protein 1	1.30E+01	1.01E+01	2.93E+01
	AT5G15490	UGD3__UDP-glucose 6-dehydrogenase family protein	3.73E+01	3.41E+01	6.37E+01
	AT1G15960	ATNRAMP6_NRAMP6__NRAMP metal ion transporter 6	1.76E+01	1.53E+01	2.86E+01
	AT3G06035	Glycoprotein membrane precursor GPI-anchored	9.40E-02	9.19E-02	9.70E-03
	AT5G54670	ATK3_KATC__kinesin 3	1.12E+00	2.23E-01	2.27E-01

	AT4G18425	Protein of unknown function (DUF679)	9.70E-02	1.92E+01	9.70E-03
	AT3G14595	Ribosomal protein L18ae family	1.03E+02	7.52E+01	5.94E+01
	AT5G59845	Gibberellin-regulated family protein	5.62E+00	1.43E+02	7.64E+00
	AT4G34320	Protein of unknown function (DUF677)	1.05E+01	5.18E+00	1.38E+01
	AT4G16620	nodulin MtN21 /EamA-like transporter family protein	1.33E+01	1.34E+00	6.65E+00
	AT2G47670	Plant invertase/pectin methylesterase inhibitor superfamily protein	9.70E-01	7.93E-02	9.70E-03
	AT3G56240	CCH__copper chaperone	2.57E+02	1.08E+03	7.17E+02
	AT1G01900	ATSBT1.1_SBTI1.1__subtilase family protein	4.07E+02	3.84E+00	2.20E+00
	AT2G25940	ALPHA-VPE_ALPHAVPE__alpha-vacuolar processing enzyme	1.06E+02	3.49E+01	3.74E+02
	AT1G05280	Protein of unknown function (DUF604)	2.52E-02	6.95E-03	0.00E+00
Biological factor-induced	AT5G38900	Thioredoxin superfamily protein	3.11E+01	1.10E+01	5.95E+01
	AT1G51890	Leucine-rich repeat protein kinase family protein	8.21E-03	2.24E-01	7.10E+00
	AT3G26830	CYP71B15_PAD3__Cytochrome P450 superfamily protein	3.15E-02	4.60E-01	5.71E+02
	AT1G13340	Regulator of Vps4 activity in the MVB pathway protein	3.35E+01	2.10E+01	4.23E+01
	AT4G33050	EDA39__calmodulin-binding family protein	2.98E+01	1.35E+01	1.59E+01
	AT1G24140	Matrixin family protein	4.62E+00	8.78E+01	3.43E+02

AT1G68620	alpha/beta-Hydrolases superfamily protein	4.83E+00	1.95E-01	5.76E-02
AT5G18470	Curculin-like (mannose-binding) lectin family protein	8.21E-03	2.52E+00	2.25E+00
AT3G63380	ATPase E1-E2 type family protein / haloacid dehalogenase-like hydrolase family protein	7.16E+01	8.49E+00	3.18E+00
AT4G36990	AT_HSFB1_ATHSF4_HSF4_HSFB1__heat shock factor 4	1.07E+01	3.75E+01	1.28E+01
AT3G47480	Calcium-binding EF-hand family protein	8.21E-03	5.97E+00	1.06E+00
AT3G22600	Bifunctional inhibitor/lipid-transfer protein/seed storage 2S albumin superfamily protein	2.37E+00	6.41E+00	4.72E+00
AT2G43570	CHI__chitinase, putative	5.71E+00	3.35E+00	5.59E+00
AT5G25930	Protein kinase family protein with leucine-rich repeat domain	5.55E+01	1.01E+00	1.72E+00
AT4G39030	EDS5_SCORD3_SID1__MATE efflux family protein	3.00E-01	6.95E-03	1.65E-01
AT1G57630	Toll-Interleukin-Resistance (TIR) domain family protein	2.21E+00	3.71E-01	4.44E-01
AT1G74590	ATGSTU10_GSTU10__glutathione S-transferase TAU 10	8.21E-03	8.27E-01	1.24E+01
AT1G65690	Late embryogenesis abundant (LEA) hydroxyproline-rich glycoprotein family	2.96E-02	6.42E+00	1.14E+01
AT2G29460	ATGSTU4_GST22_GSTU4__glutathione S-	3.43E-01	4.83E-01	6.11E-01

		transferase tau 4			
	AT1G33030	O-methyltransferase family protein	1.73E+01	6.38E+01	1.17E+01
	AT5G13080	ATWRKY75_WRKY75__WRKY DNA-binding protein 75	7.41E-01	9.43E+01	9.80E+01
	AT3G13910	Protein of unknown function (DUF3511)	3.27E+01	2.11E+02	2.13E+02
	AT1G15520	ABCG40_ATABCG40_ATPDR12_PDR12_ pletiotropic drug resistance 12	6.92E-02	6.95E-03	0.00E+00
Osmotic pressure-induced	AT1G60190	AtPUB19_PUB19__ARM repeat superfamily protein	3.52E+00	2.41E+00	4.95E+01
	AT5G66400	ATDI8_RAB18__Dehydrin family protein	8.67E-01	6.33E+00	9.70E-03
	AT2G47770	ATTSPO__TSPO(outer membrane tryptophan-rich sensory protein)-related	4.40E+01	1.07E+02	5.12E+02
	AT5G50360	unknown protein	1.16E+00	1.83E+00	1.28E+00
	AT4G24960	ATHVA22D_HVA22D__HVA22 homologue D	6.04E+00	3.00E+00	4.16E-01
	AT1G52690	LEA7__Late embryogenesis abundant protein (LEA) family protein	1.76E+00	4.35E+00	9.70E-03
	AT5G06760	AtLEA4-5_LEA4-5__Late Embryogenesis Abundant 4-5	1.85E+00	4.64E+00	9.70E-03
	AT1G07430	HAI2__highly ABA-induced PP2C gene 2	2.58E+01	8.35E+00	1.93E+01
	AT1G69260	AFP1__ABI five binding protein	5.65E+01	1.06E+01	4.74E+01
	AT5G59220	HAI1_SAG113__highly ABA-induced PP2C gene 1	3.32E+01	3.09E+00	1.37E+00

	AT2G37870	Bifunctional inhibitor/lipid-transfer protein/seed storage 2S albumin superfamily protein	1.46E+00	2.02E+03	5.34E-01
	AT2G30550	alpha/beta-Hydrolases superfamily protein	8.76E+01	1.67E+02	3.14E+01
	AT2G41190	Transmembrane amino acid transporter family protein	6.30E+00	3.80E+00	2.52E-02
	AT1G58360	AAP1_NAT2__amino acid permease 1	4.85E+00	2.62E+02	5.24E+00
	AT4G33550	Bifunctional inhibitor/lipid-transfer protein/seed storage 2S albumin superfamily protein	1.24E+00	6.20E+00	9.09E+00
	AT3G61890	ATHB-12_ATHB12_HB-12__homeobox 12	4.88E+00	6.95E-03	0.00E+00
	AT2G46680	ATHB-7_ATHB7_HB-7__homeobox 7	3.63E+01	1.35E-01	4.09E+00
	AT4G27410	ANAC072_RD26__NAC (No Apical Meristem) domain transcriptional regulator superfamily protein	2.63E+00	1.87E+02	5.11E+02
	AT5G57050	ABI2_AtABI2__Protein phosphatase 2C family protein	3.13E+01	4.74E+00	1.06E+01
	AT1G20450	ERD10_LTI29_LTI45__Dehydrin family protein	1.00E+02	6.39E+01	2.48E+01
	AT4G26080	ABI1_AtABI1__Protein phosphatase 2C family protein	3.13E+01	4.74E+00	0.35
Genotoxicity-induced	AT3G07800	Thymidine kinase	3.35E+00	1.45E+00	2.38E+00

AT5G03780	TRFL10__TRF-like 10	8.63E+00	2.35E+00	3.93E+00
AT4G22960	Protein of unknown function (DUF544)	2.85E+01	7.94E+00	2.13E+01
AT4G02390	APP_ATPARP1_PARP1_PP__poly(ADP-ribose) polymerase	3.30E+01	3.21E+00	3.41E+00
AT5G20850	ATRAD51_RAD51__RAS associated with diabetes protein 51	4.74E+00	1.61E+00	2.98E+00
AT5G48720	XRI_XRI1__x-ray induced transcript 1	1.59E+01	1.57E+00	1.30E+00
AT4G21070	ATBRCA1_BRCA1__breast cancer susceptibility1	5.93E+00	4.00E-01	1.85E+00
AT1G13330	AHP2__Arabidopsis Hop2 homolog	3.43E+00	7.89E-01	1.01E+00
AT2G18600	Ubiquitin-conjugating enzyme family protein	3.23E+01	6.73E+00	1.72E+01
AT5G24280	GMI1__gamma-irradiation and mitomycin c induced 1	3.54E+00	4.65E-01	9.73E-01
AT5G11460	Protein of unknown function (DUF581)	5.59E+01	1.57E+00	2.36E+01
AT2G45460	SMAD/FHA domain-containing protein	8.18E+00	1.53E+00	3.82E+00
AT5G64060	anac103_NAC103__NAC domain containing protein 103	1.04E+01	6.65E-01	1.83E+00
AT3G42860	zinc knuckle (CCHC-type) family protein	0.00E+00	0.00E+00	0.00E+00
AT3G27060	ATTSO2_TSO2__Ferritin/ribonucleotide reductase-like family protein	1.42E+00	6.95E-03	1.57E-01
AT4G29170	ATMND1__Mnd1 family protein	2.43E+01	1.01E+01	6.42E+00
AT5G40840	AtRAD21.1_SYN2__Rad21/Rec8-like family protein	8.88E+00	4.02E-01	1.72E+00

AT5G02220	SIAMESE/SIAMESE-RELATED (SIM/SMR) class of cyclin-dependent kinase inhibitors	1.66E+00	2.41E+01	5.62E+00
AT4G28950	ARAC7_ATRAC7_ATROP9_RAC7_ ROP9__RHO-related protein from plants 9	4.37E+00	1.62E-01	2.31E-01
AT5G67460	O-Glycosyl hydrolases family 17 protein	9.61E+00	9.36E-02	9.70E-03
AT3G20490	unknown protein	9.80E+00	2.82E+00	3.48E+00
AT5G60250	zinc finger (C3HC4-type RING finger) family protein	1.58E+01	9.70E+00	2.48E+01
AT1G17460	TRFL3__TRF-like 3	3.61E+01	5.74E+00	1.38E+01
AT4G35740	ATRECQ3_RecQl3__DEAD/DEAH box RNA helicase family protein	2.77E+00	3.69E-01	5.42E-01
AT5G55490	ATGEX1_GEX1__gamete expressed protein 1	7.25E-02	2.03E-01	8.71E-02
AT5G23910	ATP binding microtubule motor family protein	4.37E-01	6.93E-02	3.05E-01
AT5G66130	ATRAD17_RAD17__RADIATION SENSITIVE 17	1.47E+01	3.41E+00	2.68E+00
AT4G19130	Replication factor-A protein 1-related	1.40E+01	3.26E+00	1.18E+01

Table S2. The formulations of cell wall digestion enzyme solution (CWD).

Item	Volume (mL) or weight (g)
Cellulase R-10	0.6 g
Macerozyme R-10	0.16 g
0.2 M 4-Morpholineethanesulfonic acid solution	4 mL
2 M KCl solution	0.4 mL
1 M Mannitol solution	24 mL
ddH_2O	10.8 mL
1 M $CaCl_2$ solution	0.4 mL
10%BSA	0.4 mL
total	40 mL

Table S3. The formulations of buffer solution (MMG).

Item	Volume (mL) or weight (g)
0.2 M 4-Morpholineethanesulfonic acid solution	0.8 mL
1 M $MgCl_2$ solution	0.6 mL
1 M Mannitol solution	24 mL
ddH_2O	10.8 mL
total	40 mL

Table S4. The formulations of cell culture solution.

Item	Volume (mL) or weight (g)
1 mM $CuSO_4$ solution	10 μL
1 M $MgSO_4$ solution	10 μL
1 mM KI solution	10 μL
0.2 M K_2HPO_4 solution	10 μL
1 M KNO_3 solution	10 μL
1 M $CaCl_2$ solution	100μL
20% Sucrose solution	1.5 mL
MMG solution	8.35 mL
total	10 mL
